# A study of the correlation between sarcopenia and cognitive impairment in older individuals over 60 years: cross-sectional and longitudinal validation

**DOI:** 10.3389/fnagi.2024.1489185

**Published:** 2024-11-27

**Authors:** Xiaohan Dong, Yichao Yu, Jiahao Li, Xinyu Chai, Wei Shan, Huiping Yan, Yifan Lu

**Affiliations:** ^1^The School of Sports Medicine and Rehabilitation, Beijing Sports University, Beijing, China; ^2^Laboratory of Sports Stress and Adaptation of General Administration of Sport, Beijing Sport University, Beijing, China; ^3^The School of Sports Coaching, Beijing Sports University, Beijing, China, Beijing, China; ^4^Key Laboratory of Sport Training of General Administration of Sport of China, Beijing Sport University, Beijing, China

**Keywords:** sarcopenia, cognitive function, aging frailty, fluency, memory

## Abstract

**Objective:**

To validate the correlation between sarcopenia and cognition, and explore cognitive subdomains affected by sarcopenia.

**Methods:**

A case–control study was designed to recruit 90 individuals aged 60 and above from June to October 2023 in the same community, all individuals meeting the inclusion criteria were categorized according to the 2019 Asian criteria for sarcopenia and divided into the sarcopenia group and non-sarcopenia group at baseline. After a 12-week follow-up recording, individuals were classified into the aggravation group and alleviation group based on the change of sarcopenia severity. Sarcopenia tests including muscle mass, calf circumference, grip strength and physical function assessment, using Montreal Cognitive Assessment (MoCA) of nine dimensions for cognitive assessment.

**Results:**

(1) There was a significant positive correlation between cognitive function and grip strength in males (*r* = 0.42, *p* < 0.05). (2) There was a moderate correlation between sarcopenia grading and MoCA score (*r* = −0.4, *p* < 0.001). (3) Individuals with sarcopenia had significantly lower MoCA total scores and sub-scores in executive function, fluency, calculation and delayed recall compared to non-sarcopenia group (*p* < 0.05). (4) After 12 weeks, the mean value of the change in fluency in the alleviation group increased by 0.33 points, while the aggravation group decreased by 0.2 points (*W* = 128, *p* < 0.05).

**Conclusion:**

There is a correlation between sarcopenia and cognitive function, individuals with sarcopenia performing poorly in overall cognition as well as refined dimensions. The degree of cognition like fluency degenerates over time with increasing severity of sarcopenia.

## Highlights

- The relationship between sarcopenia and cognition is reflected in physical mobility and brain function.
- Cognitive function is impaired in older adults with sarcopenia.
- Some dimensions of cognition can change as muscle atrophy worsens.
- The slowing speed caused by sarcopenia could be reflected in cognitive expression.

## 1 Introduction

Sarcopenia is an aging-related, progressive and generalized skeletal muscle loss disorder characterized by decreased muscle mass, decreased muscle strength, and/or reduced somatic functions (Cesari and Kuchel, 2020), and it is associated with frailty (Gielen et al., 2023), heart disease (Sasaki and Fukumoto, 2022), diabetes (Guerrero et al., 2016; Umegaki et al., 2017), and many other chronic conditions. The definition and diagnosis of sarcopenia continues to evolve, in 2019 the Asian Working Group for Sarcopenia (AWGS) proposed defining muscular dystrophy as “sarcopenia” as well (Chen et al., 2020), which means that sarcopenia is no longer a disease in the traditional sense, but rather a state of muscle atrophy that accompanies aging. Similarly, cognitive decline is a pathological condition that includes a range of cognitively related symptoms. Frailty is a multidimensional syndrome including physical, social, and cognitive aspects, among which physical and cognitive decline are fundamental dimensions (Arai et al., 2018; Kelaiditi et al., 2013).

Current findings in Asia and Europe have found that sarcopenia was associated with the incidence of neurological disorders such as cognitive dysfunction, Alzheimer’s disease and depression (Yang et al., 2022; Lee et al., 2018), and was even a predictor of Alzheimer’s disease, mild cognitive impairment (MCI) and cognitive decline (Beeri et al., 2021). Sarcopenia and cognitive dysfunction not only share many common pathogenic mechanisms of myokines, endocrine and inflammatory markers, but also have some external influencing factors such as lifestyle of lack of physical activity and poor nutritional habits (Cannataro et al., 2021; Sui et al., 2020). Evidence suggests that muscle loss increases the risk of cognitive decline, such as reduced handgrip strength and gait speed (Basile and Sardella, 2021; Chou et al., 2019).

The diagnosis of sarcopenia in early stages has positive implications for the prevention of cognitive dysfunction (Peng et al., 2020). The presence of early motor dysfunction has been suggested as a potential predictor of further cognitive impairment, such as the motoric cognitive risk syndrome (MCR)(Basile and Sardella, 2021). At the same time, separate diagnostic indicatorss of sarcopenia have been associated with cognitive dysfunction (McGrath et al., 2020). Better cognition was associated with a lower risk of developing sarcopenia and could explain some of the potential pathways contributing to sarcopenia (Hu et al., 2024). Studies in the US, Spain, Brazil, Saudi Arabia, and Japan reported a range of MCI prevalence between 6.5 and 38.6% (Lu et al., 2021), compared with 20.8% reported by the Chinese National Centre for Prevention and Control of Chronic and Non-communicable Diseases (Jia et al., 2014), that early recognition of cognitive deterioration is essential to prevent further deterioration of cognitive impairment, especially to prevent MCI turning into dementia. A study based on the China Health and Retirement Longitudinal Study (CHARLS) showed that the incidence of MCI in non-sarcopenia, possible sarcopenia, and sarcopenia groups was 10.1%, 16.5%, and 24.2%, this not only confirms the cross-sectional association between the two, but also suggests the progression of muscle atrophy will accelerate cognition decline.

Although several studies have been conducted to assess potential associations, most of them focused on cognition in general and did not analyze subdomains in detail, and previous studies have demonstrated significant differences in age (Hu et al., 2022; Salinas-Rodriguez et al., 2021; Sugimoto et al., 2022). Considering whether sarcopenia contributes to cognitive decline, we delve into the cognitive profiles of individuals with sarcopenia, with particular emphasis on specific cognitive domains. Subsequently, we assess the evolution of muscle atrophy and cognitive abilities over a 12-week period to explore whether the process of muscle atrophy is accompanied by cognitive function decline.

## 2 Methods

### 2.1 Study population

The study population was recruited in Yanda Retirement Community in June 2023, Langfang, Hebei, China. Inclusion criteria: (1) aged 60 and above; (2) education level of college and above; (3) participation in annual health checkups; (4) clear consciousness without communication barriers; (5) reasonable medication and stable physical condition; (6) having signed an informed consent form. Exclusion criteria: (1) patients with serious diseases or multiple diseases; (2) severe neurological disorders such as dementia; (3) unable to move independently; (4) hospitalized or exit during the experiment.

### 2.2 Study design

**Figure 1:**
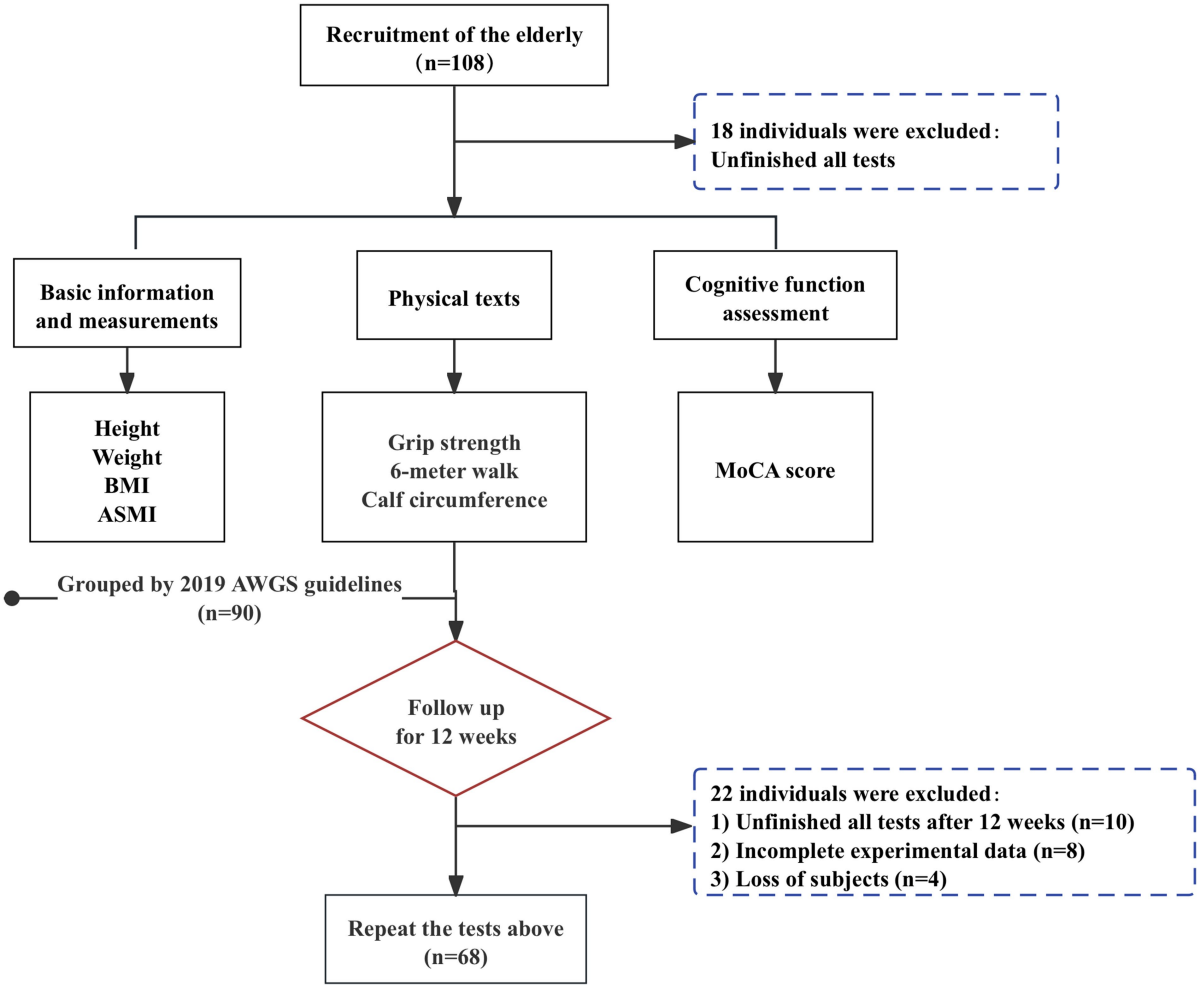
Flowchart of the experiment.

At baseline, case–control was used to classify individuals into non-sarcopenia and sarcopenia groups, consisting of 90 individuals aged 60 to 95, including 43 males and 57 females. Followed by a 12-week follow-up intervention in which all older adults participated in the exercise program to varying degrees and recorded, and were classified into the aggravation group and the alleviation group, based on the change in the progression of sarcopenia relative to baseline after 12 weeks. A total of 108 people were surveyed at baseline and 90 completed the entire process and recorded their performance, and 68 completed the recording after 12 weeks. The detailed flow chart of the sample selection is shown in Figure 1. The study protocol was approved by the Sport Science Ethics Committee of Beijing Sport University (No. 2020082H).

### 2.3 Measurement

#### 2.3.1 Screening for sarcopenia

Sarcopenia was evaluated according to the AWGS guidelines, including calf circumference, muscle strength, skeletal muscle mass (ASM) and physical performance (Chen et al., 2020).

Calf circumference was measured using a long tape measure. Muscle mass of the upper and lower extremities was obtained by Inbody (230, Korea), and the total skeletal muscle mass of the extremities was summed to obtain the appendicular skeletal muscle mass index (ASMI), which was calculated by the formula: total skeletal muscle mass of the extremities (kg)/height^2^ (m^2^). ASMI below 7.0 kg/m^2^ in male and 5.7 kg/m^2^ in female in community were considered as low muscle mass.

Grip strength was assessed using a grip strength meter (Jamar Inc., USA) tested with individuals in a standing position using the dominant hand and squeezing the grip strength meter with maximum force 2 times at 1-min intervals, the maximum values were chosen. Grip strength below 28 kg for male and 18 kg for female was considered low muscle strength.

Somatic mobility function was assessed using the 6-m walking tests, which required subjects to walk along a straight with step speed of on their usual, and the time taken to walk 6 m was recorded by a stopwatch to calculate the average speed. The test was performed twice and the minimum value was included. A 6-m walking speed of less than 1.0 m/s was considered as low physical performance.

**Figure 2:**
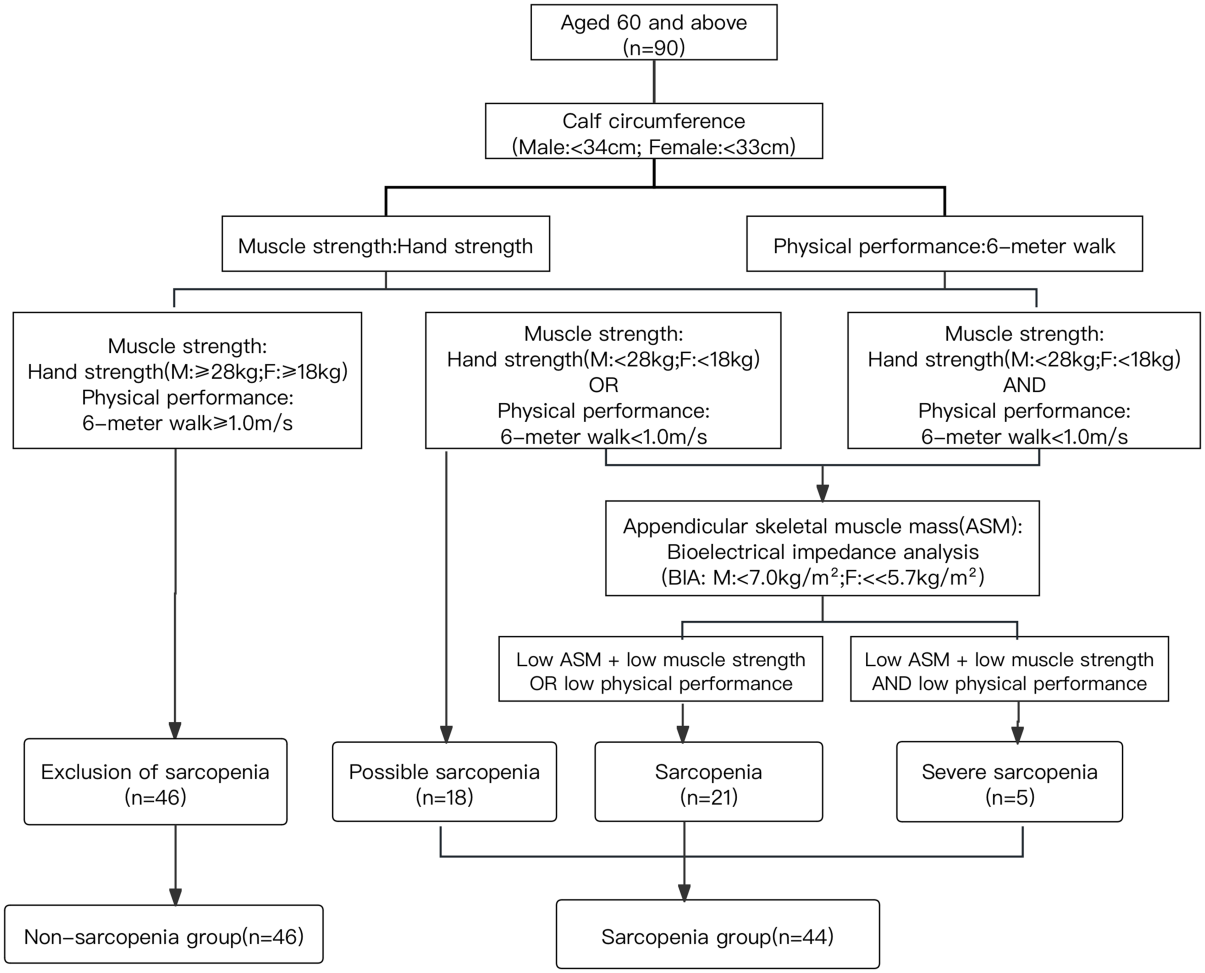
Screening groupings for sarcopenia.

Accordingly, the sarcopenia group was categorized into 3 parts. The group of possible sarcopenia was defined as low muscle strength or low physical performance, the group of sarcopenia was defined as low muscle mass and low muscle strength/low physical performance, the group of severe sarcopenia was defined as low muscle mass, low muscle strength and low physical performance (Chen et al., 2020). The screening groupings for sarcopenia are shown in Figure 2.

#### 2.3.2 Cognitive function assessment

The MoCA was used to assess the cognitive functioning of the older. Nine domains including executive function, fluency, orientation, calculation, abstract thinking, delayed recall, visuospatial functions, naming and attention. The full scale value is 30, ≥26 being normal, 18–25 being MCI and 10–17 being moderate cognitive impairment, and less than 10 being severe cognitive impairment. The MoCA is an alternative to the MMSE (MiniMental State Examination), but compared to the MMSE, it was more consistent with the screening test standards for MCI in the older over 60 (Ciesielska et al., 2016), and also easier to detect the risk of dementia in patients with cognitive impairment (Dong et al., 2012). A study in Chinese middle-aged and older population also proves that ceiling effect for MCI was less frequent using MoCA versus MMSE (Jia et al., 2021). Overall, the MoCA is a better choice for screening cognitive impairment in community-dwelling older people.

#### 2.3.3 Potential covariates

Considering sociodemographic characteristics and health-related factors in our study. Sociodemographic characteristics included age and gender, and other relevant factors collected through questionnaires included acute inflammation in 3 months, alcohol consumption, and common chronic diseases of the older (hypertension, hyperlipidemia, diabetes, cancer, respiratory diseases, liver diseases, heart disease, stroke, urinary system diseases, digestive system diseases, nervous system diseases, and bone and joint diseases). Height and weight were measured using standardized procedures, and body mass index (BMI) was calculated as weight (kg) /height^2^ (m^2^). The diagnostic criteria for obesity were based on the Chinese and World Health Organization (WHO) standards. According to the Chinese BMI classification (Pan et al., 2021; Zhou, 2002), underweight was defined as <18.5 kg/m^2^, normal weight was 18.5 to <24 kg/ m^2^, overweight was 24 to <28 kg/m^2^, and obesity was ≥28 kg/m^2^.

### 2.4 Statistical analysis

Data processing was performed using the RStudio (version 4.3.1). Quantitative data that satisfied normality were expressed as mean ± standard deviation, and independent samples *t*-tests were used for between-group comparisons; quantitative data that were not normal were expressed as median [interquartile spacing] and nonparametric tests were used for between-group comparisons. For categorical variables the individual number (percentage) was used for representation and chi-square test was used for comparison between groups. Spearman correlation analysis was used for correlation analysis between MoCA score and severity of sarcopenia. The effect sizes of the correlation coefficient *r* were classified as below: < 0.25 for weak correlation, 0.25–0.5 for moderate correlation, 0.5–0.75 for strong correlation, and **≥** 0.75 for extremely strong correlation, the statistical significance was set at *p* < 0.05.

## 3 Results

### 3.1 General data on baseline measurements of study subjects

Table 1 shows the baseline data of the population. There was no difference in basic information about age, gender, weight and BMI between the non-sarcopenia and sarcopenia groups, and the height of the older was 160.4 ± 7.1 cm in the non-sarcopenia group and 155.7 ± 7.6 cm in the sarcopenia group (*p* = 0.003). The total MoCA cognitive score was 27.5 [26.3, 28.8] in the non-sarcopenia group, and 25.0 [24.0, 27.0] in the sarcopenia group, which was a significant difference between the two groups (*p* < 0.001). The cognitive assessment included 9 domains of executive function, fluency, orientation, calculation, abstract thinking, delayed recall, visuospatial functions, naming and attention. The baseline basic measurements of the study subjects are shown in Table 1.

**Table 1:** Baseline essential measurements.

Item		Level	Non-sarcopenia group	Sarcopenia group	*p*-value
			(*n* = 46)	(*n* = 44)	
Sarcopenia grade, *n* (%)		Non-sarcopenia	46 (100%)	0 (0%)	
		Possible	0 (0%)	18 (40.9%)	
		Sarcopenia	0 (0%)	21 (47.7%)	
		Severe	0 (0%)	5 (11.4%)	
Age (year), M [P25,P75]			82.0 [78.3, 86.8]	84.5 [81.0, 88.0]	0.059
Gender, *n* (%)		Male	17 (37.0%)	16 (36.4%)	0.961
		Female	29 (63.0%)	28 (63.6%)	
Height (cm), M ± SD			160.4 ± 7.1	155.7 ± 7.6	0.003
Weight (kg), M ± SD			61.1 ± 9.5	59.5 ± 9.3	0.425
BMI (kg/m^2^), M ± SD			23.7 ± 2.9	24.5 ± 3.1	0.222
Grip strength (kg), M ± SD			25.2 ± 5.3	18.6 ± 5.6	<0.001
Calf circumference (cm), M [P25,P75]			33.0 [31.7, 35.8]	33.0 [31.6, 35.1]	0.478
6-meter pace (m/s), M [P25,P75]			0.3 [0.2, 0.3]	0.4 [0.3, 0.4]	<0.001
ASMI (kg/m^2^), M ± SD			6.5 ± 0.1	6.1 ± 0.1	0.033
MoCA score, M [P25,P75]			27.5 [26.3, 28.6]	25.0 [24.0, 27.0]	<0.001
Cognitive level, *n* (%)		Normal	38 (82.6%)	19 (43.2%)	<0.001
		MCI	8 (17.4%)	21 (47.7%)	
		Moderate	0 (0%)	4 (9.1%)	
Obesity (kg/m^2^), *n* (%)		Underweight (<18.5)	0 (0%)	2 (4.5%)	0.406
		Normal (18.5 to <24)	28 (60.9%)	20 (45.5%)	
		Overweight (24 to <28)	15 (32.6%)	16 (36.4%)	
		Obese (≥28)	3 (6.5%)	6 (13.6%)	
Acute inflammation in 3 months, *n* (%)		No	35 (76.1%)	37 (84.1%)	0.493
		Yes	11 (23.9%)	7 (15.9%)	
Chronic diseases	Alcohol consumption, *n* (%)	No	39 (84.8%)	36 (81.8%)	0.925
		Yes	7 (15.2%)	8 (18.2%)	
	Chronic diseases, *n* (%)	No	22 (47.8%)	20 (45.5%)	0.989
		Yes	23 (50.0%)	24 (54.5%)	
	Hyperlipidemia, *n* (%)	No	24 (52.2%)	33 (75.0%)	0.319
		Yes	17 (37.0%)	11 (25.0%)	
	Diabetes mellitus, *n* (%)	No	33 (71.7%)	35 (79.5%)	0.538
		Yes	13 (28.3%)	9 (20.5%)	
	Cancer, *n* (%)	No	45 (97.8%)	42 (95.5%)	0.969
		Yes	1 (2.2%)	2 (4.5%)	
	Respiratory diseases, *n* (%)	No	42 (91.3%)	40 (90.9%)	1.000
		Yes	4 (8.7%)	4 (9.1%)	
	Liver diseases, *n* (%)	No	45 (97.8%)	43 (97.7%)	1.000
		Yes	1 (2.2%)	1 (2.3%)	
	Cardiovascular diseases, *n* (%)	No	33 (71.7%)	32 (72.7%)	1.000
		Yes	13 (28.3%)	12 (27.3%)	
	Stroke, *n* (%)	No	41 (89.1%)	41 (93.2%)	0.761
		Yes	5 (10.9%)	3 (6.8%)	
	Urinary diseases, *n* (%)	No	45 (97.8%)	40 (90.9%)	0.331
		Yes	1 (2.2%)	4 (9.1%)	
	Digestive diseases, *n* (%)	No	40 (87.0%)	39 (88.6%)	1.000
		Yes	6 (13.0%)	5 (11.4%)	
	Neurological diseases, *n* (%)	No	45 (97.8%)	40 (90.9%)	0.331
		Yes	1 (2.2%)	4 (9.1%)	
	Bone and joint diseases, *n* (%)	No	27 (58.7%)	24 (54.5%)	0.854
		Yes	19 (41.3%)	20 (45.5%)	
	Other diseases, *n* (%)	No	35 (76.1%)	34 (77.3%)	1.000
		Yes	11 (23.9%)	10 (22.7%)	

BMI, body mass index; ASMI, appendicular skeletal muscle mass index; MCI, mild cognitive impairment; MoCA score, testing scores of Montreal cognitive assessment; M ± SD, mean ± standard deviation; M [P25, P75], median [25th percentile, 75th percentile]; *n* (%), individual number (percentage).

### 3.2 Relationship between cognitive function and diagnostic indicators of sarcopenia

**Figure 3:**
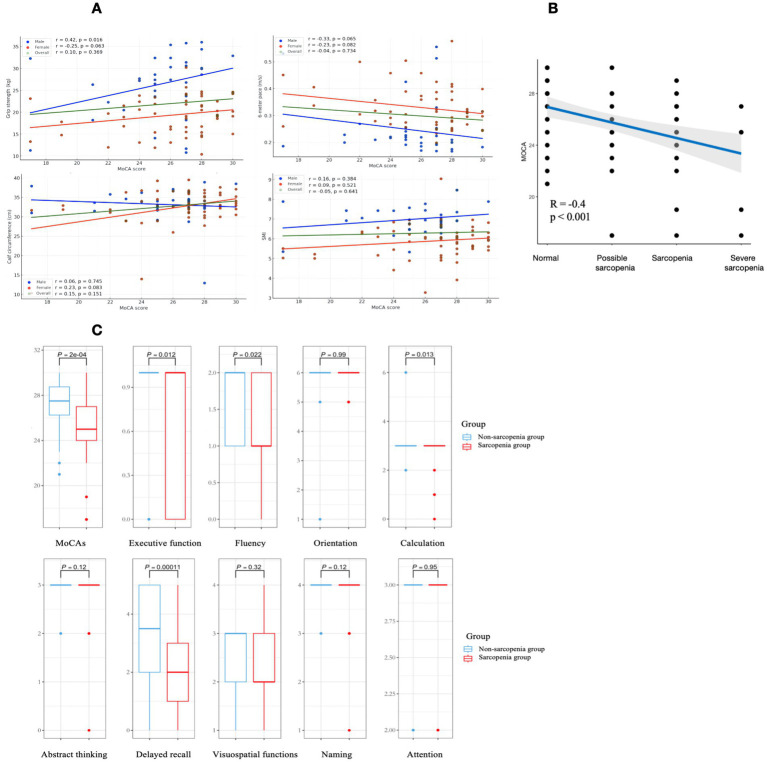
Graphical representation of data analysis images are labeled (A–C). (A) Demonstrates the relationship between cognitive function and diagnostic indicators of sarcopenia in the total population, males and females. Green, blue, and red are used to represent the overall population, males and females respectively. (B) Demonstrates the variation and correlation of the total MoCA score with those with different degrees of sarcopenia, with a significant negative correlation. (C) Demonstrates the differences between sarcopenia and healthy elderly on the cognitive subitem, with sarcopenia group performing worse on the executive function, fluency, calculation and delayed recall.

The total population did not show a significant correlation. But in males, there was a significant positive correlation between cognitive function and grip strength (*r* = 0.42, *p* < 0.05), as shown in Figure 3A, no significant correlation between cognitive function and 6-m step speed (*p* = 0.065), calf circumference (*p* = 0.74) and SMI (*p* = 0.38). In females, there was no significant correlation between cognitive function and grip strength (*p* = 0.063), 6-m step speed (*p* = 0.082), calf circumference (*p* = 0.083) and SMI (*p* = 0.52).

### 3.3 Differences in cognitive function with different sarcopenia degrees

The total MoCA scores of individuals with different degrees of sarcopenia were further analyzed. According to the guidelines of AWGS of Figure 2, the non-sarcopenia group remained unchanged (*n* = 46), the population in the sarcopenia group was subdivided into possible sarcopenia (*n* = 18), sarcopenia (*n* = 21) and severe sarcopenia (*n* = 5). Compared to the non-sarcopenia group, the MoCA score progressively decreased with decreasing muscle mass and increasing muscle atrophy. There was a moderate correlation between the grade of sarcopenia and MoCA score (*r* = −0.4, *p* < 0.001), as shown in Figure 3B, indicating that individuals with severe sarcopenia are more likely to experience cognitive dysfunction.

### 3.4 Relationship between sarcopenia and cognitive dimension

Figure 3C shows the differences in terms of the total cognitive score and the 9 subitems. The MoCA scores of the sarcopenia and non-sarcopenia groups were 27.5 [26.3, 28.7] and 25.0 [24.0, 27.0] respectively, the MoCA scores were lower in the sarcopenia group (*p* < 0.05). Among the 9 subitems, the scores of 4 subitems of executive function, fluency, calculation and delayed recall in the sarcopenia group had a proportion of low scores in the distribution, with statistically significant differences (*p* < 0.05). The differences in orientation, abstract thinking, visuospatial perception, naming and attention were not statistically significant in the sarcopenia group relative to the non-sarcopenia group (*p* > 0.05).

### 3.5 Cognitive deterioration in the progression of muscle atrophy

**Figure 4:**
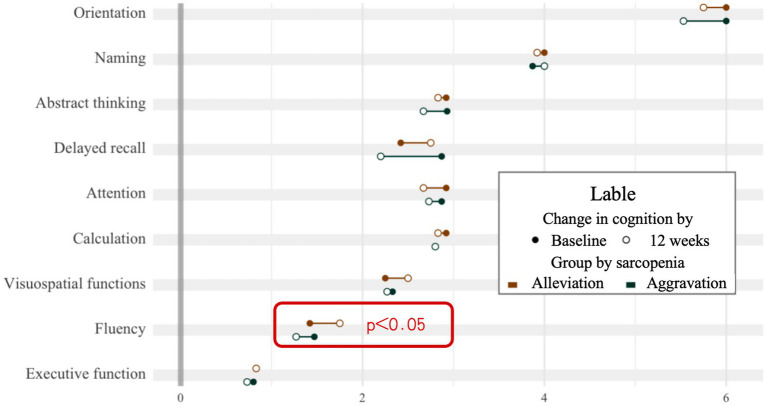
Changes in cognitive subdomains (baseline to 12 weeks).

After 12 weeks of naturalistic observation, there was a change in the degree of sarcopenia and cognitive functioning, the two groups were analyzed for potential influencing factors such as age, level of physical activity, as shown in Table 2. According to the 4 grades grouping of Figure 2, those who showed a progressing severity of muscle atrophy at the end of the 12 weeks were considered as the aggravation group (*n* = 15), and those who showed a relieving in grading was the alleviation group (*n* = 12). Although the two groups did not show a significant difference in the total MoCA scores, there was a significant change in the cognitive subdomains of fluency, as shown in Figure 4. The mean value of the change in fluency for alleviation group was an increase of 0.33 points, whereas the change of aggravation group was a decrease of 0.2 points, which was a statistically significant difference (*p* = 0.038).

**Table 2:** Longitudinal information for the aggravation group and alleviation group.

	Level	Alleviation group (*n* = 12)	Aggravation group (*n* = 15)	*p* value
Level of physical activity, *n* (%)	1/week	4 (33.3%)	4 (26.7%)	0.446
	2/week	2 (16.7%)	4 (26.7%)	
	3/week	5 (41.7%)	3 (20.0%)	
	4/week	1 (8.3%)	4 (26.7%)	
Age, M ± SD		83.8 ± 5.5	86.5 ± 4.1	0.143
Gender, *n* (%)	Male	4 (33.3%)	7 (46.7%)	0.759
	Female	8 (66.7%)	8 (53.7%)	
Height, M ± SD		156.1 ± 5.8	157.4 ± 10.2	0.708
Weight, M ± SD		59.4 ± 4.5	58.3 ± 11.3	0.753
BMI (kg/m^2^), M ± SD		24.5 ± 3.0	23.4 ± 3.3	0.363
Degree of muscle atrophy at baseline, *n* (%)	Non-sarcopenia	0 (0%)	11 (73.3%)	0.001
	Possible	5 (41.7%)	0 (0%)	
	Sarcopenia	6 (50.0%)	4 (26.7%)	
	Severe	1 (8.3%)	0 (0%)	
Degree of muscle atrophy at 12 weeks, *n* (%)	Non-sarcopenia	9 (75.0%)	0 (0%)	0.001
	Possible	2 (16.7%)	7 (46.7%)	
	Sarcopenia	1 (8.3%)	3 (20.0%)	
	Severe	0 (0%)	5 (33.3%)	
Baseline MoCA, M ± SD		25.7 ± 2.3	25.7 ± 2.9	0.948
12-week MoCA, M [P_25_,P_75_]		26.5 [25.0, 27.3]	25.0 [23.5, 27.5]	0.376
Difference of MoCA, M [P_25_,P_75_]		0.5 [−1.0, 2.0]	0 [−2.5, 1.0]	0.302

BMI, body mass index; MoCA score, testing scores of Montreal cognitive assessment; M ± SD, mean ± standard deviation; M [P25, P75], median [25th percentile, 75th percentile]; *n* (%), individual number (percentage).

## 4 Discussion

Through a cross-sectional and longitudinal study, we validated the correlation between sarcopenia and cognitive function, revealing changes in several cognitive subdomains. We demonstrated a moderate positive relationship between cognitive scores and grip strength in older males, suggesting grip strength as a reliable predictor of cognitive impairment. However, no significant differences were observed with calf circumference and muscle mass. Individuals with sarcopenia exhibited lower total MoCA cognitive, particularly in executive function, fluency, calculation and delayed recall. Cognitive ability further declined with increasing severity of muscle atrophy. The progression of muscle atrophy was associated with significant deterioration in cognitive functions, such as fluency.

More research confirmed that cognitive function was related to muscle strength rather than muscle mass (Moon et al., 2016; Menant et al., 2017), while cross-sectional studies have shown an association between overall cortical atrophy and low skeletal muscle mass in patients with cognitive impairment (Abellan van Kan et al., 2012). Both the AWGS and the Chinese Expert Consensus emphasize grip strength as an important indicator for screening and diagnosing sarcopenia (Chen et al., 2020). Grip strength was reliable and consistent with MCI and dementia, making it a useful tool for exploring the sarcopenia-cognition relationship (McGrath et al., 2020; Dudzińska-Griszek et al., 2017). A systematic evaluation revealed that six out of seven studies documented a significant correlation between grip strength, hand dexterity, and cognitive function in older adults (Kobayashi-Cuya et al., 2018). Recent research by Vancampfort et al. (2019) reported that lower grip strength in middle-aged and older adults was associated with an increased likelihood of MCI. Others have shown correlations between grip strength and cognitive subdomains such as MMSE (Dudzińska-Griszek et al., 2017), information processing speed and executive function (Hooghiemstra et al., 2017), stroop task performance and 6-item cognition test scores (Ramnath et al., 2018). This suggests that delaying cognitive decline is just as important as maintaining physical mobility. Therefore, we suggest that perhaps more attention should be paid to muscle work and daily mobility than to musculature.

The worsening of sarcopenia has been found to lead to poorer cognitive functioning, particularly in specific dimensions. Previous studies indicate that Chinese elderly with more severe sarcopenia have higher rates of MCI compared to those with possible sarcopenia (Hu et al., 2022). These findings suggest that the severity of sarcopenia increases the likelihood of cognitive impairment and negatively impacts cognitive function. Analyses from the CHARLS database showed associations between sarcopenia and cognitive functions such as orientation, memory, calculation and drawing (Hu et al., 2022). Other studies also reported correlations between sarcopenia and verbal fluency and recall abilities (Salinas-Rodriguez et al., 2021; Szlejf et al., 2019). Similar to these results, fluency and delayed recall were also significantly worse in our results, and we believe that fluency is associated with recall and expression, which explains why dementia and Alzheimer’s disease occur at higher rates in people with sarcopenia (Beeri et al., 2021; Waite et al., 2021). Computational ability and logical agility were related to thought integration, it is thought that people with sarcopenia have reduced responsiveness and poorer executive performance, explaining their motor speed such as poor gait speed and decreased balance (Bahureksa et al., 2017).

The association between sarcopenia and cognitive impairment could be explained from physiological and functional links. Neuromuscular junction dysfunction (Moreira-Pais et al., 2022), neuronal hyperexcitability, dopaminergic dysfunction, muscle-brain axis (Arosio et al., 2023) and brain atrophy are among the regulatory processes associated with the pathophysiology of sarcopenia. Secondly, changes in the structure and function of the neuromuscular system. A prospective study in 2021 found moderate to severe parietal lobe atrophy, overall cortical atrophy, and medial temporal lobe atrophy were more common in the sarcopenia group compared to the non-sarcopenia group (Hsu et al., 2021). In the same year, a Korean longitudinal study involving 1,284 individuals over four years using magnetoencephalography found a greater decrease in gray matter volume in the sarcopenia group (Yu et al., 2021). This finding suggests that sarcopenia accelerates gray matter atrophy, low muscle mass is linked to reduced frontal, parietal, and occipital gray matter volumes, with significant parietal gray matter atrophy in sarcopenic patients. Finally, in terms of functional performance, older individuals with sarcopenia are more prone to falls as they experience a decline in balance, in turn the poorer the muscle control and balance of body, less active and more susceptible to sarcopenia, producing a slower gait speed (Kim and Won, 2019), decreased balance (Basile and Sardella, 2021), and psycho-emotional functioning (Lee et al., 2018; Ohta et al., 2019; Scisciola et al., 2021).

Although the causal relationship have not been confirmed, most studies have shown that the deterioration of physical function precedes cognitive function decline, such as gait speed and suffering from MCR (Basile and Sardella, 2021; Skillbäck et al., 2022). Therefore, strengthen physical activity are commended, which will not only improve muscle performance (Billot et al., 2020), but also affect cognition through the muscle-brain axis, such as L-6, which increases 100-fold during physical exercise (Pedersen and Febbraio, 2008). Post-exercise blood circulation also regulates brain functions and protects neurons from damage, increases neurogenesis and plasticity in the hippocampus, enhances the function of the prefrontal cortex (Scisciola et al., 2021; Nuzum et al., 2020). These can also be maintained through nutritional supplementation, creatine monohydrate supplementation may confer beneficial effects on cognitive function particularly in the domains of memory, attention time, and processing speed (Xu et al., 2024), Vitamin D and the Mediterranean diet can also help prevent dementia when providing the body with adequate nutrition (Cannataro et al., 2021; Psaltopoulou et al., 2013). We propose that sarcopenia aggravates cognitive impairment, lifestyle factors such as exercise and diet may improve the progressive aggravation.

Several limitations are in this study. First, although age and chronic diseases have been controlled, the baseline habitual physical activity of the participants was not assessed. In this study, only diagnostic indicators of sarcopenia were evaluated, using scales for cognitive function rather than objective indicators, such as brain imaging. Second, the causal relationship between muscle atrophy and cognitive impairment is difficult to explain. Last, the number of samples retained during the secondary collection was small. These need to be further refined in subsequent studies.

## 5 Conclusion

There is a correlation between sarcopenia and cognitive function, with older individuals in sarcopenia performing worse in overall cognition as well as in executive function, fluency, calculation and delayed recall. Muscle atrophy was accompanied by cognitive decline after the exclusion of intervening factors such as age, chronic diseases and physical activity, particularly affecting verbal fluency. Sarcopenia and decreased muscle function may have dual implications for the exacerbation of cognitive impairment.

## Data Availability

The raw data supporting the conclusions of this article will be made available by the authors, without undue reservation.
